# Food purchase diversity is associated with market food diversity and diets of children and their mothers but not fathers in rural Tanzania: Results from the EFFECTS baseline survey

**DOI:** 10.1111/mcn.13734

**Published:** 2024-10-24

**Authors:** Savannah F. O'Malley, Ramya Ambikapathi, Morgan Boncyk, Dominic Mosha, Cristiana K. Verissimo, Lauren Galvin, Frank Mapendo, Isaac Lyatuu, Mary Pat Kieffer, Joshua Jeong, Evidence Matangi, George PrayGod, Nilupa S. Gunaratna

**Affiliations:** ^1^ Department of Nutrition Science Purdue University West Lafayette Indiana USA; ^2^ Department of Public Health Purdue University West Lafayette Indiana USA; ^3^ Department of Global Health The George Washington University; ^4^ Department of Global Development Cornell University Ithaca New York USA; ^5^ Department of Health Promotion, Education, and Behavior University of South Carolina Columbia South Carolina USA; ^6^ Department of Health Epidemiology division St. Louis Missouri USA; ^7^ Department of Global Health BeVera Solutions LLC Riverdale GA USA; ^8^ Purdue Extension Nutrition Education Program Lebanon Indiana USA; ^9^ Department of Program Quality and Accountability Global Communities 8601 Georgia Ave #300 Silver Spring Maryland USA; ^10^ Gender Equality Section UNICEF New York USA; ^11^ Research and Program Unit Africa Academy for Public Health Dar es Salaam Tanzania; ^12^ Department of Global Health and Population Harvard T.H. Chan School of Public Health Boston Massachusetts USA; ^13^ Hubert Department of Global Health Emory University Atlanta Georgia USA; ^14^ Mathematics Department Taylor University Upland Indiana USA; ^15^ Muhimbili Research Centre National Institute for Medical Research Dar es Salaam Tanzania

**Keywords:** dietary diversity, food environment, food purchases, market access, market food diversity, Tanzania, young children

## Abstract

Rural households in East Africa rely on local markets, but the influence of market food diversity and household food purchase diversity on diets has not been well‐characterized. We quantify the associations among market food diversity, household food purchase diversity and dietary diversity of mothers, fathers and children in rural Tanzania. This study uses baseline data from a randomized controlled trial, Engaging Fathers for Effective Child Nutrition and Development in Tanzania. We used the 10 food groups for women's dietary diversity to assess the seasonal availability of nutritious foods in 79 markets. Using data from 957 rural households in two districts in Mara, Tanzania, we measured household food purchase diversity over the previous month and dietary diversity among children (6–23 months), mothers and fathers. Overall, 63% of markets sold all 10 food groups throughout the year, indicating high‐market food diversity and minimal seasonality. However, only 33% of women and 35% of children met dietary diversity recommendations. Households that reported higher purchasing power (0.14, *p* < 0.001), lived within 30 min of a market (0.36, *p* = 0.001) and had access to a highly diverse market (0.37, *p* = 0.01) purchased a higher diversity of foods. In turn, food purchase diversity was positively associated with the dietary diversity of mothers (*p* < 0.001) and children 9–23 months (*p* < 0.001) but not fathers (*p* = 0.56). Interventions must account for food availability and access in local markets, and promoting diverse food purchases may be an effective strategy to improve women's and children's diets in rural areas.

## Key messages

- Local markets in rural Mara, Tanzania, had unexpectedly high food diversity with minimal seasonality.
- Physical and economic access to diverse food markets was associated with households' food purchase, which was in turn associated with women's and children's consumption of diverse diets.
- Fathers' diets were more diverse and not associated with the diversity of food purchases for consumption at home.
- The metric developed here—household food purchase diversity—captures food diversity that could contribute to nutrient intake of children and adults.
- Intervention design must account for the broader food environment in rural settings.

## Graphical abstract

We quantified the association between market food diversity, household food purchase diversity and the diets of mothers, fathers and children in rural Tanzania. Market food diversity was associated with higher food purchase diversity, which in turn was associated with improved dietary diversity for mothers and children but not fathers.

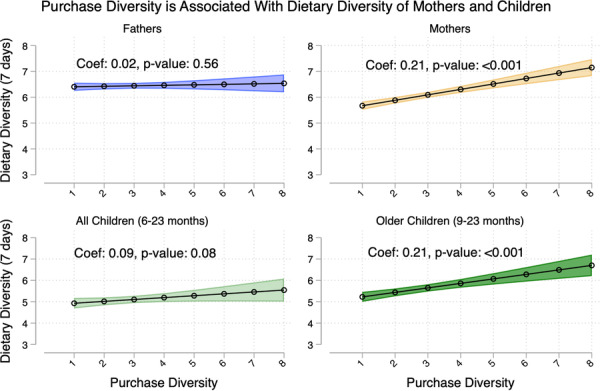

## 1 INTRODUCTION

Undernutrition remains a substantial challenge throughout the world, particularly in low‐ and middle‐income countries (LMIC). Around 30% of people in East Africa were undernourished in 2019, totalling more than 136 million (FAO et al., 2022). In Tanzania, over 34% of children under 5 years are stunted, and 58% of children aged 6–59 months are anaemic (Ministry of Health, C. D., Gender, Elderly and Children‐MoHCDGEC/Tanzania Mainland, Ministry of Health ‐ MoH/Zanzibar, National Bureau of Statistics ‐ NBS/Tanzania, Office of Chief Government Statistician ‐ OCGS/Zanzibar, & ICF, 2016). Dietary diversity indicates dietary quality and is closely associated with nutrition outcomes for children and women (Ruel et al., 2013). Only 26% of children 6–23 months in Tanzania are consuming the minimum dietary diversity, and rural children are less likely to consume diverse diets than urban children (Ministry of Health, C. D., Gender, Elderly and Children‐MoHCDGEC/Tanzania Mainland, Ministry of Health—MoH/Zanzibar, National Bureau of Statistics—NBS/Tanzania, Office of Chief Government Statistician ‐ OCGS/Zanzibar, & ICF, 2016).

The food environment is defined as ‘the interface that mediates people's food acquisition and consumption within the wider food system’, shaping the diets of households and individuals (Turner et al., 2018, p. 95). In rural East Africa, foods are available from two primary sources: household production and local markets (Hirvonen & Hoddinott, 2017; Koppmair et al., 2016). Many studies have focused on diversifying home production as the pathway to improving women's and children's diet quality, but increasing home production is generally associated with only small shifts in dietary diversity (Jones, 2017), and a recent meta‐analysis reported that agricultural interventions do not improve child dietary diversity in the absence of a behaviour change component (Margolies et al., 2022). Additionally, a recent review of 20 studies found that market access was positively associated with dietary diversity in many contexts, including East Africa (Nandi et al., 2021). Although markets are a promising pathway to improving dietary diversity, there are substantial gaps in the published literature. Very little is known about key market characteristics, such as availability of foods in markets (Headey et al., 2019). To our knowledge, only two studies have quantified the association between market food diversity (MFD) and dietary diversity. In rural Ethiopia, the diversity of nonstaple (nutrient‐dense) food items available in the market was associated with increased child consumption of nonstaple foods, although the effect size was small (Headey et al., 2019). Ambikapathi et al. reported that access to markets with greater food diversity had varied impacts on the diets of women in Ethiopia, depending on the local ecology (Ambikapathi et al., 2019). These results indicate that the availability of foods in local markets (a dimension of the food environment) is associated with diets, but the mechanisms of this association are not established in the literature.

We hypothesize that MFD positively influences food purchase diversity and, ultimately, individual dietary diversity. However, we are unaware of any studies that have quantified the association between MFD and food purchase diversity. The first part of our analysis quantifies this association. Next, we quantify the association between food purchase diversity and dietary diversity in three populations (young children, their mothers and their fathers). To our knowledge, only two studies have quantified the association between food purchase diversity and dietary diversity. In Benin, the number of food items purchased and consumed by the mother was associated with women's dietary diversity (Bellon et al., 2016). In Malawi, the increased diversity of foods purchased was positively associated with household dietary diversity score (Matita et al., 2021). We are not aware of any studies that have quantified the association between household food purchase diversity and the dietary diversity of other individuals, such as young children or fathers. To address these gaps, we utilize cross‐sectional data from 957 households in rural Tanzania, utilizing a novel metric for food purchase diversity, which uses the same 10 food groups as dietary diversity for women (FAO & FHI 360, 2016), and a metric for MFD similar to studies in Ethiopia (Ambikapathi et al., 2019) and rural Rufiji, Tanzania (Madzorera et al., 2021).

## 2 METHODS

### 2.1 Population

This paper draws data from the baseline evaluation of the Engaging Fathers for Effective Child Nutrition and Development (EFFECTS) trial (ClinicalTrials.gov Identifier: NCT03759821) in Butiama and Musoma districts in Mara, Tanzania. Mara is a dry region in northwest Tanzania, bordering Lake Victoria. Growing crops, raising livestock and fishing are primary livelihoods for this population (EFFECTS Study Investigators, 2024). The protocol for the study has been published elsewhere (EFFECTS Study Investigators, 2024). Briefly, the EFFECTS study is a cluster‐randomized controlled trial that engaged fathers and mothers to improve nutrition and early childhood development outcomes among young children. Depending on the intervention arm, mothers and fathers participated in peer groups to improve knowledge and practices around infant and young child feeding, water and sanitation, couples' communication and parenting. Sessions were held biweekly for 24 sessions (approximately 12 months). The primary outcomes were child dietary diversity and early childhood development. Intervention effects on child development (Jeong et al., 2024) and gender equality and women's empowerment (Galvin et al., 2023) have been published elsewhere. Households (*n* = 960) were randomly sampled from 80 villages. A household was eligible if there resided a child 0–18 months at the time of enrolment, along with the child's mother and father for at least 10 months out of the year, and if the household was planning to stay in the area during the EFFECTS study. In intervention villages, households were included if mothers (and fathers, when appropriate) were willing to participate in peer groups for the intervention delivery. The cross‐sectional data for this study was collected before the interventions in two stages. Most data collection occurred in both Musoma and Butiama in December 2018–February 2019. However, due to administrative challenges, 107 mothers and five markets were surveyed in May of 2019. Of the 107 mothers, 80 (75%) lived in Musoma. Due to some delay between enrolment and baseline data collection, children's age at baseline was between 0 and 24 months. All questionnaires used in the study were piloted before data collection with mothers and fathers of children of the target age group living in the study area but not enroled in the study.

### 2.2 MFD

Markets were assessed for seasonal availability, sales volume, prices (Headey et al., 2019) and sources of 69 commonly consumed food and beverage items over the last 12 months. At each market, one respondent, such as a trader or village leader, was identified to recall market data over the preceding 12 months. MFD (Ambikapathi et al., 2019) was scored in the following manner: one point was assigned for the presence of each of the 10 food groups that contribute to Dietary Diversity. The 69 food items were grouped into the 10 food groups that contribute to Minimum Dietary Diversity for Women (MDD‐W) to compare market diversity to dietary diversity and household purchase diversity. Items such as rock salt and sugar, which did not contribute to any food group, were excluded. One point was assigned for each month that the food groups were sold in the market the previous year, resulting in a score between 0 (indicating no food groups sold at any point in the year) and 120 (indicating that all 10 food groups were present for each month of the year). For ease of interpretation, an average MFD score was calculated by dividing the overall score by 12 to indicate the average availability of food groups in each month over the last year. Therefore, the average MFD score was on a scale from 0 to 10.

### 2.3 Household food purchasing

Food purchase patterns were calculated from a questionnaire that queried mothers about household‐level consumption and sources of 19 food groups. If that food group was consumed, the mothers were asked what was the main source (own production, market, fishing/gathering, received as a gift, received in exchange for labour, food aid and others). The food groups were designed to be similar to the designations for MDD‐W (FAO & FHI 360, 2016), with differentiation between some types of foods (e.g., chicken eggs and other eggs, chicken meat and other meat). These 19 food groups were (i) grains, (ii) other starchy foods, (iii) pulses, (iv) nuts and seeds, (v) dark green leafy vegetables, (vi) orange flesh fruits, (vii) other vegetables, (viii) other fruits, (ix) milk and milk products, (x) chicken meat and organ meat, (xi) other organ meat, (xii) other meat and poultry, (xiii) fish and seafood, (xiv) chicken eggs, (xv) other eggs, (xvi) red palm oil, (xvii) other oils, (xviii) savoury and fried snacks and (xix) sweets. The first 15 of these 19 food groups were used to calculate the diversity of household foods from market purchases that contribute to MDD‐W so that household purchase diversity would be on the same scale as dietary diversity (0–10). The last four groups (red palm oil, other oils, savoury snacks and sweets) were excluded from the calculation of purchase diversity because they did not contribute to dietary diversity or because the group contributed to multiple food groups (in the case of sugary and fried snacks which were presumed to be comprised mostly of staples, sugar and oil). In addition to purchasing behaviours, mothers were asked about market attendance and minutes to market.

### 2.4 Household access to food markets

Household physical access to markets was measured as the mother's self‐reported minutes to market (usually walking), and household economic access to markets was measured by purchasing power (total yearly household expenditures, reported by fathers). Purchasing power was calculated from the recall of more frequent expenditures that occurred over the previous month (the sum of food, fuel, transport, communication, entertainment, utilities, personal care, monthly savings and other frequent expenditures) multiplied by 12, plus less frequent expenditures occurring over the previous year (agriculture, clothes and shoes, education expenses, social events, housing improvement, human health, yearly savings, loans and taxes).

### 2.5 Dietary diversity

Father, mother and child diets were measured using a food consumption questionnaire that queried 24‐h and 7‐day consumption of 96 food items. The field team helped to develop this questionnaire to capture data on the most commonly consumed foods. Consumption of the 96 food items was aggregated to report dietary diversity for men and women scored based on consumption of 10 food groups as described by the MDD‐W (FAO, & FHI 360, 2016). The 10 food groups were (i) staples, (ii) pulses, (iii) seeds and nuts, (iv) dairy), (v) flesh foods, (vi) eggs, (vii) dark green leafy vegetables, (viii) vitamin A‐rich produce, (ix) other vegetables and (x) other fruits. We created a binary variable for having consumed at least 5 of the 10 food groups in the previous 24 h. The same variable was calculated for women and men.

We computed dietary diversity for children 6–23 months of age (*n* = 764), as outlined by the World Health Organization (WHO, 2008). The seven food groups were (i) staples, (ii) legumes and nuts, (iii) dairy, (iv) flesh foods, (v) eggs, (vi) vitamin A‐rich produce and (vii) other fruits and vegetables. We focus on complementary foods which could be sourced from the market; thus, we do not consider breast milk consumption. Minimum dietary diversity is defined as consuming four or more of the seven food groups in the previous 24 h. Children 0–5 months were excluded from this analysis because it is recommended that mothers exclusively breastfeed their children until 6 months of age.

The final analysis predicted child dietary diversity over the previous 7 days computed in 10 food groups to mirror the 10 food groups for dietary diversity for women, listed above. For example, the group (vi) ‘vitamin A‐rich produce’ for child dietary diversity was split into two groups to match dietary diversity for women: ‘dark green leafy vegetables’ and ‘other vitamin A‐rich fruits and vegetables’. A child consuming both amaranth leaves and ripe mango would have received a score of 1 according to dietary diversity for children, as both amaranth leaves and ripe mango are grouped in the ‘vitamin A‐rich produce’ category but would have received a score of 2 using the 10 food groups scoring because amaranth leaves are a ‘dark green leafy vegetable’ while ripe mangoes are ‘other vitamin A‐rich fruits and vegetables’. Groundnuts were mistakenly omitted, although they are a part of local diets (Verissimo, 2022).

### 2.6 Statistical analysis

We examined associations between MFD, household food purchase patterns and consumption of food groups among men, women and children aged 6–23 months. These analyses are exploratory. Sample size and power calculations for the EFFECTS study are available in the published protocol (EFFECTS Study Investigators, 2024). Multi‐level mixed effects linear and logistic regression models were used to evaluate the relationship between key exposures (MFD, market access, economic access) and outcomes (purchase diversity and odds of purchasing specific food groups) while adjusting for clustering at the village level. In the final analyses, we used a multi‐level effects linear regression model to evaluate the relationship between purchase diversity and dietary diversity of men, women and children. We analyzed the association between purchase diversity and child dietary diversity for children 6–23 months. Next, we repeated this analysis for a subset of older children (9–23 months) because these older children likely had diets more reflective of adults (Verissimo, 2022). For our final regression models, we ran models with imputed missing values for yearly purchasing power and number of household members for the 47 fathers who were missing from baseline collection. Categorical data with missing values (caregiver education and wealth index) included a category for missing information. For bivariate models between purchase diversity and purchasing power and household members, we included an indicator variable for households with imputed values. A variable for the mother's survey group (i.e., December–February vs. May) was added to the models to account for seasonal changes over the period of data collection. Regression models predicting purchase diversity included both unadjusted (bivariate) and multi‐variable (adjusted) analyses, each accounting for clustering at the village level. The regression models predicting child dietary diversity included whether the child had been breastfed the previous day (data for *n* = 743 children aged 6–23 months). Although the data was collected as part of the EFFECTS study, we used baseline data here, before the intervention began. Therefore, we do not include the study arm in the analysis. The district was not added to the model due to its association with both groups of data collection and MFD. All analyses were performed in STATA16. Results were considered significant at *p* < 0.05.

### 2.7 Ethics statement

This study was conducted according to the guidelines laid down in the Declaration of Helsinki, and all procedures involving research study participants were approved by the ethics committees at Harvard University (with which Purdue has a reliance agreement), Project Concern International (now Global Communities) (which also ceded to Harvard University) and the National Institute for Medical Research, Tanzania. Written informed consent was obtained from all subjects, and caregivers provided consent for the children.

## 3 RESULTS

### 3.1 Demographics

Data was collected from 957 women, 913 men and 764 children aged 6–23 months, of which 597 children were aged 9–23 months. The median household size was seven (Q1–Q3: 5–8) members (Table 1). The median age of children was 11 months (Q1–Q3: 7–17). Education among household heads was fairly homogenous, as 77% of fathers and 81% of mothers reported completing grade seven (primary school). The median time to the nearest food market was 30 min. A typical household owned two acres (Q1–Q3:1–4). Most households (92%) reported consuming foods in the last month that were produced at home, which contributed three food groups to dietary diversity. The foods most commonly grown at home were vitamin A‐rich fruits and vegetables, staples and produce not rich in vitamin A. Eighty‐five percent of households owned chickens (Median: five birds, Q1–Q3: 2–10), while 42% owned cattle and 45% owned goats. Seventy‐three percent of households reported moderate or severe food insecurity in the preceding month (Table 1).

**Table 1:** Demographics of population (*n *= 957).

	Median (Q1, Q3) or %
Household size	7 (5, 8)
Age of child (months)	11 (7, 17)
Father age (years)	37 (31, 45)
Mother age (years)	29 (24, 35)
Father years of education	
No formal education	3.0
Did not complete primary school	6.9
Completed primary school	72.2
Completed some secondary/tertiary school	11.9
Missing info	6.0
Mother years of education	
No formal education	5.1
Did not complete primary school	4.3
Completed primary school	76.1
Completed some secondary/tertiary school	7.5
Missing info	7.0
Self‐reported minutes to market	30 (10, 60)
Yearly expenditures, million TSH (*n *= 913)	2 (1, 3)
Total land owned (*n *= 913)	2 (1, 4)
Does not own land (*n *= 913)	13.9
Number of food categories (1–10) that a household produced	3 (2, 4)
Access to electricity	8.1
Running water in the home (*n *= 913)	1.1
Access to safe water^a^	33.4
House wall made of improved materials^b^ (*n *= 913)	40.0
House roof made of improved materials^c^ (*n* = 913)	79.7
House floor made of improved materials^d^ (*n *= 913)	22.1
Food security (HFIAS status) at baseline	
Food secure	12.9
Mildly food insecure	14.6
Moderately food insecure	23.0
Severely food insecure	49.5

*Note*: Q1, first quartile. Q3, third quartile.

Abbreviation: HFIAS, household food insecurity access scale; TSH, Tanzanian Shillings.

^a^ Safe water refers to households that boiled water or treated with bleach/chlorine, waterguard, pur, or aquatabs, used biosand/composite/ceramic filter or used solar disinfection.

^b^ Improved wall denotes concrete, bricks or corrugated metal as opposed to rudimentary wall (mud, cardboard, etc.), traditional wall (stones) or walls made from palm, bamboo, straw or leaves.

^c^ Improved roof denotes roof made from metal or concrete, as opposed to thatched roofs.

^d^ Improved floor denotes cement, concrete, wood, bamboo or finished (such as tile, ceramic or mosaic), as opposed to floor made with mud.

### 3.2 Household food purchase patterns

Overall, households most commonly purchased flesh foods (87% of households), staples (60%) and dark green leafy vegetables (43%) (Table 2). For some foods (e.g., staples), percent of households purchasing is less than percent of individuals consuming that food group, indicating that some households acquired staples from home production or other sources.

**Table 2:** Purchase diversity of households in the last month (*n *= 957) and diets of men (*n *= 913), women (*n *= 957) and children aged 6–23 months (*n *= 764) during the previous day.

	Median (Q1, Q3) or %			
	Household purchasing	Fathers' consumption	Mothers' consumption	Child (6–23 months) consumption
**Diversity indicator**	***N *= 957**	***N *= 913**	***N *= 957**	***N *= 764**
% meeting MDD^a^	‐	42.3	32.8	35.9
Diversity^b^	3 (2, 4)	4 (3, 5)	4 (3, 5)	3 (2, 4)
Staples	59.5	99.9	99.4	90.6
Legumes	29.6	22.9	22.5	15.6
Nuts	12.6	1.1	0.8	0.5
Dairy	19.6	31.4	31.9	34.8
Flesh foods	86.8	73.1	63.4	49.0
Eggs	0.8	3.7	2.8	1.8
Dark green leafy vegetables	36.6	38.4	33.0	20.2
Other vitamin A‐rich produce	22.3	57.1	49.8	30.0
Other vegetables	12.4	80.8	76.6	58.4
Other fruits	3.2	26.7	20.4	12.7

Abbreviation: MDD, Minimum Dietary Diversity.

^a^ MDD, an indicator whether an adult has consumed five or more of 10 food groups, or whether a child 6–23 months has consumed four or more of seven food groups in the past 24 h.

^b^ Diversity is purchase diversity over 1 month (for household purchasing column), dietary diversity out of 10 food groups in 24 h, according to the MDD‐W (Fathers' Consumption and Mothers' Consumption columns) and dietary diversity out of the standard seven food groups in 24 h for children 6–23 months (last column). Note that prevalence of consumption is higher than prevalence of purchasing (e.g., for staple foods), indicating that some households acquire staple foods from other sources such as home production.

### 3.3 MFD

In contrast to the poor DD within households and low household food purchase diversity, market surveys revealed that 50 of 79 markets (63%) sold all 10 food groups contributing to MDD‐W year‐round, with little overall seasonal variation in food item or food group availability (Figure 1a,b). All markets sold grains, seeds, flesh foods and other vegetables all year. Fifteen markets did not sell eggs at all. Mangoes, which are rich in vitamin A, were highly seasonal (Figure 1b). All markets sold candy, soda, biscuits and *chapatis* (thin fried bread, frequently consumed outside the home). MFD was associated with the district, as more than 75% of markets in Butiama had high MFD (10 food groups year‐round), while only 51% of markets in Musoma had high MFD (Figure 1).

Figure 1Location of 79 surveyed villages and their market food diversity scores (a) and seasonal availability of food items (b). High‐diversity markets are indicated in orange. These markets are reported to sell all 10 food groups each month of the year. Base map data © 2023 Google.

**Figure:**
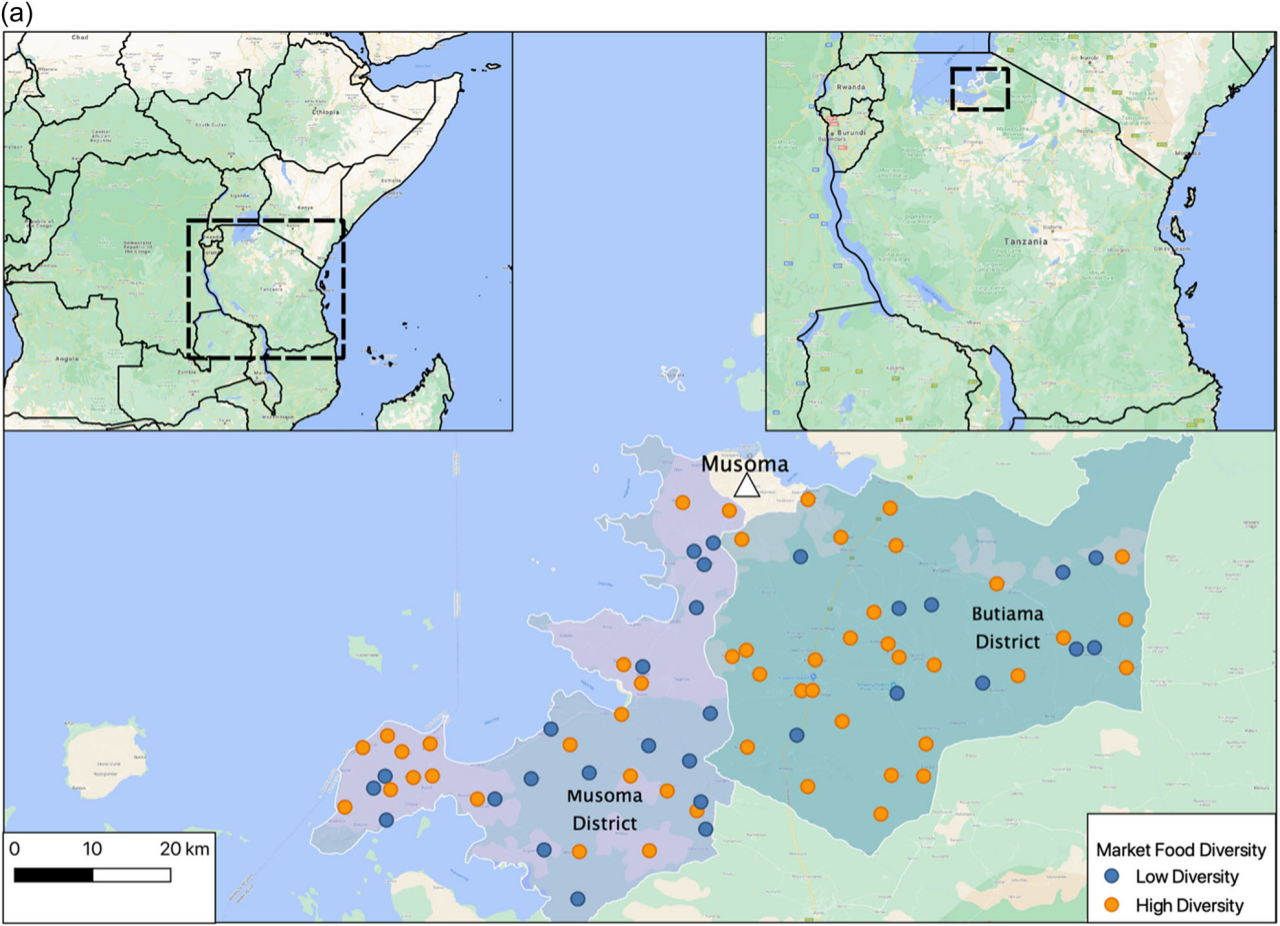

**Figure:**
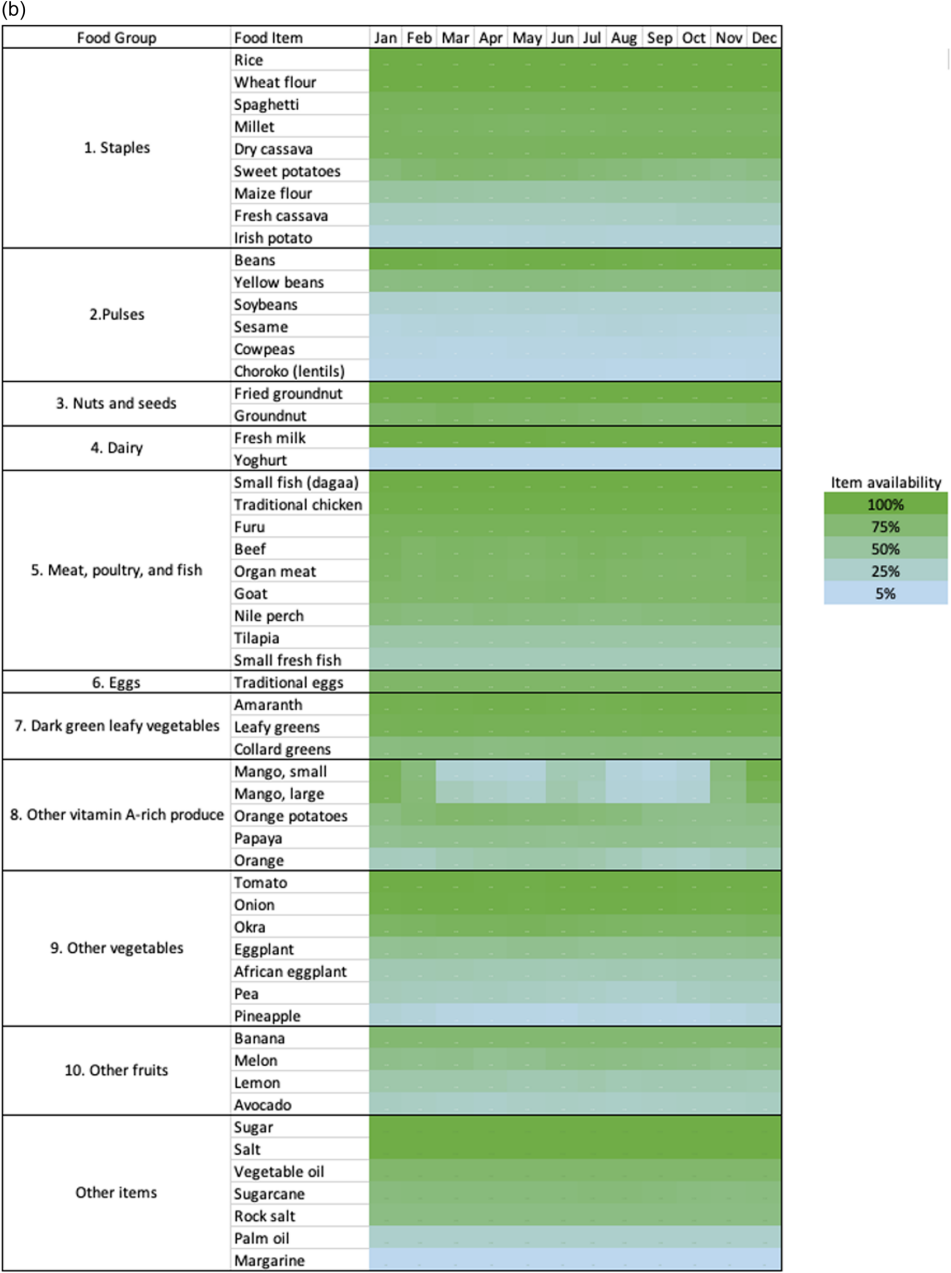

### 3.4 Associations between purchase diversity and the food environment

Although the majority of households had access to highly diverse markets, mothers' reports of household food purchase patterns did not reflect the diversity of foods available in markets. Median purchase diversity over the previous month was three food groups (Q1–Q3: 2–4; Table 2). In the unadjusted bivariate models, living within 30 walking min of a market and having access to a highly diverse market was associated with purchasing 0.73 more food groups (*p *< 0.05; Table 3). In addition, economic access was associated with improved purchase diversity. Households in the higher quartile of wealth had increased purchase diversity compared to poorest households. Similarly, an increase in spending of a million Tanzanian shillings (approximately $430) per year by the household was associated with purchasing 0.14 more food groups (*p *< 0.001). In the adjusted model, proximity to a market, wealth quartile and yearly expenditures remained significant predictors, but MFD was marginally significant (*p *= 0.08; Table 3).

**Table 3:** Predictors of household food purchase diversity (*n* = 956).

	Unadjusted model			Adjusted model		
	Estimate	SE	*P* Value	Estimate	SE	*P* Value
Household within 30 walking min of a market	0.36	0.11	0.001	0.32	0.10	0.002
High‐market food diversity^a^	0.37	0.15	0.01	0.25	0.14	0.08
Household in the lowest asset quartile	Ref.			Ref.		
Household in the second asset quartile	0.14	0.15	0.35	0.07	0.14	0.64
Household in the third asset quartile	0.15	0.15	0.32	0.05	0.15	0.72
Household in the fourth asset quartile	0.34	0.15	0.02	0.17	0.15	0.24
Household in the highest asset quartile	0.29	0.15	0.06	0.02	0.16	0.91
Missing	0.63	0.24	0.01	−0.72	0.67	0.28
All household expenditures during the year, in millions of Tanzanian Shillings^b^	0.14	0.03	<0.001	0.12	0.03	<0.001

Abbreviation: SE, standard error.

^a^ High‐market food diversity indicates that the household has access to a market which sells all ten food groups for 12 months out of the year.

^b^ Denotes variable with imputed values for 57 missing fathers; value imputed from the mean of 913 fathers. Models accounting for imputed values did not differ significantly. Unadjusted model controlled for clustering at the village level. Adjusted model controlled for time of survey (December 2018 to February 2019 vs. May 2019), maternal education, household size, including a dummy variable missing household size.

Multi‐level logistic regression results show that being within 30 min of a market was positively associated with the odds of purchasing dark green leafy vegetables (odds ratio [OR] 1.49, 95% confidence interval [CI]: 1.09–2.05), other Vitamin A‐rich produce (OR 1.75, 95% CI: 1.09–2.80) and staples (OR 1.53, 95% CI: 1.11–2.10) (Supporting Information S1: Table 1). Yearly household purchasing power was associated with the odds of purchasing pulses (OR 1.13, 95% CI: 1.02–1.25), and vegetables not rich in Vitamin A (OR 1.15, 95% CI: 1.01–1.30). High MFD is associated with purchasing staples (OR 1.56, 95% CI: 1.06–2.32). Eggs and other fruits are not reported in Supporting Information S1: Table 1, as less than four percent of households reported purchasing these food groups in the previous month.

### 3.5 Diets of women, men and children

Dietary diversity was poor among women, men and children, although men had more diverse diets than women and children (Table 2). Only 33% of women consumed the minimum recommended dietary diversity of five out of 10 food groups in the last 24 h (FAO & FHI 360, 2016). In comparison, 43% of men consumed five out of 10 food groups, and 35% of children aged 6–23 months consumed the recommended minimum of four out of seven food groups (WHO, 2008). An average adult diet consisted of staples (e.g., maize, wheat, rice and sorghum), produce (generally onions and tomatoes), animal source foods such as fish relish and vitamin A‐rich produce (e.g., amaranth leaves, pumpkin leaves and mangoes). A higher percentage of men reported consumption of each food group than women and children, with the exception of the dairy group, of which children consumed more than either mothers or fathers.

### 3.6 Associations between purchase diversity and dietary diversity of women, men and children

**Figure 2:**
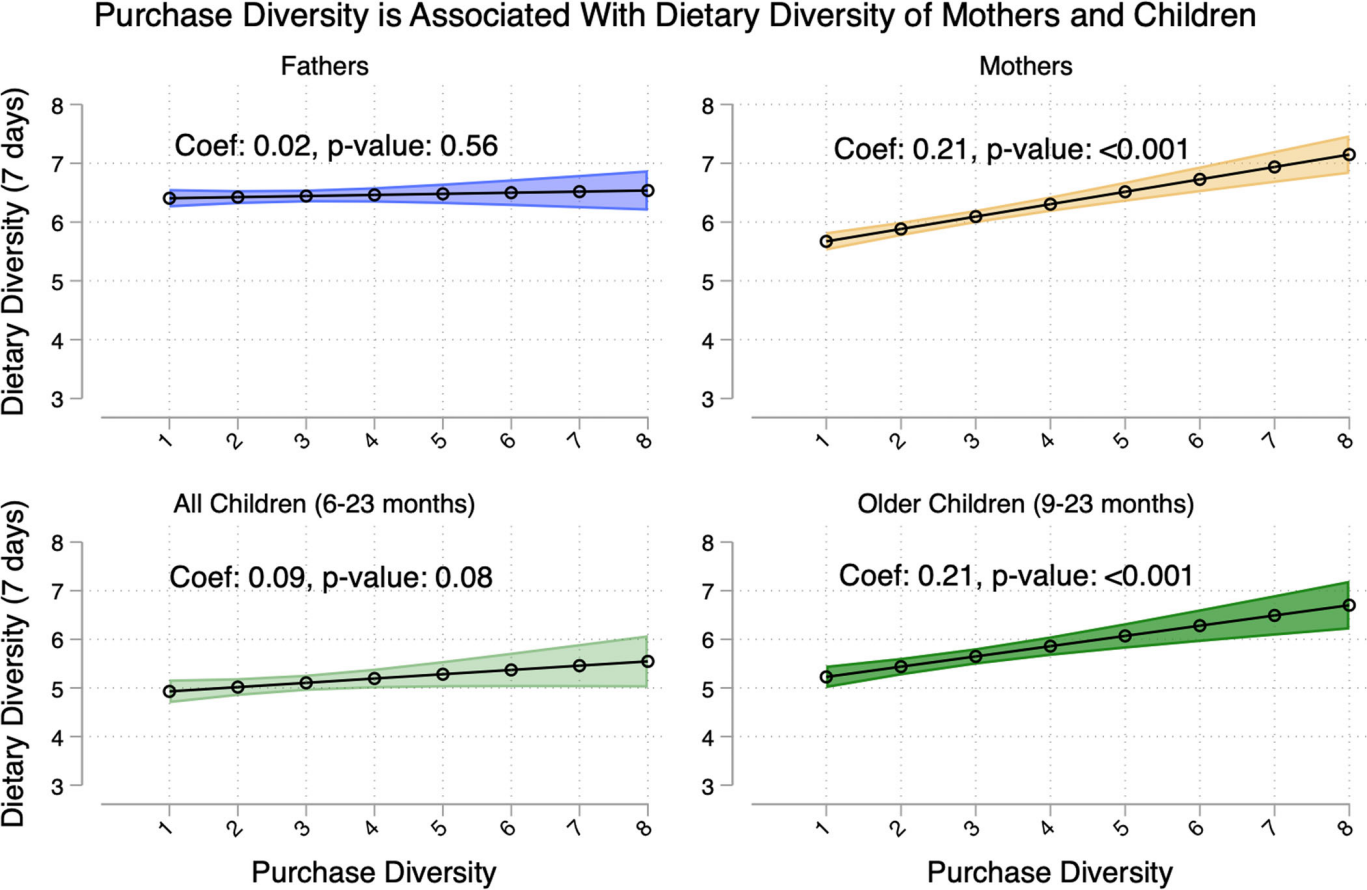
Household food purchase diversity is associated with improved dietary diversity among mothers and older children. All dietary diversity scores are measured across 7 days using 10 food groups. Purchase diversity was associated with dietary diversity among mothers (b = 0.21, *p* < 0.001, *n *= 957), among children 6–23 months (b = 0.09, *p* = 0.081, *n *= 743) and among children aged 9–23 months (b = 0.21, *p* < 0.001, *n *= 586). Purchase diversity was not significantly associated with fathers' dietary diversity (*p* = 0.56, *n *= 913). Models adjust for time of data collection, living within 30 min of a market, market food diversity, yearly expenditures and household wealth and size. The analyses for children adjust for whether the child was breastfed the previous day. The analysis for fathers adjusted for the father's education, while the analyses for mothers and children adjusted for the mother's education.

We found that overall purchase diversity was significantly associated with dietary diversity for mothers ($\hat{\beta }$ = 0.21, *p* < 0.001) but not fathers ($\hat{\beta }$ = 0.02, *p* = 0.56) (Figure 2). The relationship between purchase diversity and dietary diversity of children is not significant ($\hat{\beta }$ = 0.09, *p* = 0.08) when all children 6–23 months were included. However, for a subset of older children (9–23 months), this relationship became more substantial and significant ($\hat{\beta }$ = 0.21, *p* < 0.001).

## 4 DISCUSSION

We found that household food purchase diversity is associated with both MFD and with dietary diversity of children and women. Household food purchase diversity was substantially lower than the diversity of foods available in the local markets but was greater among households that had access to a market with high food diversity. In turn, household food purchase diversity was positively associated with improved dietary diversity for children and mothers but not with dietary diversity for fathers. These findings concur with the existing literature that local markets are an important source of nutritious foods for rural households. To our knowledge, this paper is the first to quantify the association between MFD and household food purchase diversity, and the first to quantify the association between food purchase diversity and dietary diversity of children and fathers. Our results strengthen the evidence that the effect of market access on diets is mediated by household purchases and intrahousehold food allocation. These findings suggest that increasing household food purchase diversity could enable more nutritious diets for children and women.

Food purchase diversity was also associated with physical access (time to market) and economic access (household purchasing power) after controlling for relevant covariates. These results are similar to a study conducted in Benin (Bellon et al., 2016) and with data from Ethiopia and Malawi (Sibhatu et al., 2015). These findings indicate that nutrition programmes must continue to be sensitive to the physical and economic barriers that households face when purchasing nutritious foods. The association between food purchase diversity and dietary diversity in our sample of rural farming households strengthens the evidence that markets contribute to diets even in rural areas. Bellon and colleagues also found that food purchases were associated with the diets of mothers (Bellon et al., 2016), while Matita and colleagues reported that purchase diversity was associated with household dietary diversity score (Matita et al., 2021). We add to this evidence by demonstrating that the relationships between purchase diversity and dietary diversity were similar between mothers and older children, but not for fathers. The lack of relationship between food purchase diversity and fathers' dietary diversity may indicate that fathers access additional sources of food, such as eating away from home, and that household foods are preferentially allocated to fathers. Ochieng et al. conducted focus group discussions with men in arid regions of Dodoma and Mbeya, Tanzania, finding that men reported frequently consuming foods outside of the home (Ochieng et al., 2017).

Overall, dietary diversity was poor in this sample. Adults consumed more diverse diets than children, indicating that child consumption was influenced by household food environment and allocation beyond household‐level access to food, as shown in formative research for the EFFECTS trial (Verissimo, 2022). Among younger children, the relationship between food purchase diversity and dietary diversity may be attenuated due to perceptions around appropriate complementary foods for children 6–8 months of age that have been reported previously (Verissimo, 2022). The diets of older children more closely reflect dietary patterns among adults as they transition to family foods over time.

This study has several important strengths. Data were collected at three levels: individual, household and market. We collected data on fathers' diets alongside mothers' and children's, thus giving insight into intrahousehold food allocation. In addition, we surveyed markets for seasonal availability of diverse foods, providing information on temporal and spatial variation in MFD. This market data collection is simple and could be implemented in other studies without adding excessive burden. By categorizing purchase food diversity and MFD using the same categories as dietary diversity for women, we can highlight the importance of purchases for dietary diversity (Ambikapathi et al., 2019). Furthermore, we include information about market participation (purchases) in addition to physical market access. Our study also has limitations. First, all data were based on recall, which likely weakened the analysis through recall bias. Information was recalled over several time periods, ranging from the previous day (diets) to monthly over the previous year (markets). The different recall periods may make comparisons between dietary diversity, household food purchase diversity and MFD more difficult. In addition, recall of MFD over the previous year may have been particularly subject to recall bias due to the long recall period and potentially by food availability at the time of the interview. If respondents had more diverse food available at the time of the interview, they may have over‐reported the availability of foods from previous months. This may explain why MFD reported in May (several months after harvest) was lower than MFD for the same months when reported in December through February (during the harvest season).

## 5 CONCLUSION

These results have important implications for future research and interventions. There is a growing recognition that food environments shape diets in low‐ and middle‐income countries (Turner et al., 2019). This work expands the limited knowledge of rural food markets (Headey et al., 2019). Purchase diversity is positively associated with dietary diversity in our results and other literature (Bellon et al., 2016; Matita et al., 2021), implying that increasing household food purchase diversity is one plausible pathway to improve dietary diversity. Nutrition interventions in rural areas may be more effective by accounting for the broader food environment and shifting focus to consider local markets alongside household production as sources of nutritious foods. For example, intervention materials can account for the seasonal availability of foods in local markets when designing recommendations for acquiring nutritious foods. Formative research should also measure additional sources of food (e.g., foods consumed away from home) and recognize that interventions along the market pathways may not equally benefit every household member if interventions do not also consider the effects of gender and intrahousehold food allocation. Such formative research is critical to designing interventions tailored to the target environment.

Several research questions remain. First, rural food environments in LMICs are poorly characterized, with little published about seasonal food availability in local markets; future research should prioritize mapping the availability of foods from diverse sources in rural contexts. Second, future research should consider additional dimensions of the food environment, including food prices, convenience and desirability (Turner et al., 2019) to identify pathways to improving dietary diversity. Third, future research should determine if it is possible to improve dietary diversity by influencing household purchasing decisions while accounting for intrahousehold gender dynamics. We recommend that food purchase diversity be formally measured and reported in a metric consistent with dietary outcomes. Surveying household food purchase diversity is a simple way to capture household reliance on markets, which can be integrated into the intervention messaging. A greater understanding of local food environments and household food purchasing diversity may enable future nutrition interventions to better tailor nutrition messaging and have a greater impact on rural diets and nutrition.

## AUTHOR CONTRIBUTIONS

*Formulation of research question*: Savannah F. O'Malley and Nilupa S. Gunaratna. *Led the analysis*: Savannah F. O'Malley, Ramya Ambikapathi and Nilupa S. Gunaratna. *Wrote the manuscript*: Savannah F. O'Malley, Ramya Ambikapathi and Nilupa S. Gunaratna. *Led implementation of the study*: Ramya Ambikapathi, Lauren Galvin and Nilupa S. Gunaratna. *Led fieldwork and data collection*: Dominic Mosha and Frank Mapendo. *Provided feedback for the manuscript*: Ramya Ambikapathi, Morgan Boncyk, Dominic Mosha, Cristiana K. Verissimo, Lauren Galvin, Frank Mapendo, Isaac Lyatuu, Mary Pat Kieffer, Joshua Jeong, Evidence Matangi, George PrayGod and Nilupa S. Gunaratna.

## CONFLICT OF INTEREST STATEMENT

The authors declare no conflict of interest.

## Supporting information

Supporting information.

## Data Availability

EFFECTS principal investigators to enquire about access to de‐identified study data.
