# Supplementary material for: Food purchase diversity is associated with market food diversity and diets of children and their mothers but not fathers in rural Tanzania: Results from the EFFECTS baseline survey
Source: Matern Child Nutr. 2024 Oct 24;21(1):e13734. doi: 10.1111/mcn.13734 (PMC11650043; doi:10.1111/mcn.13734)
Supplement: Supplementary file 1 — Supporting information. [file MCN-21-e13734-s001.docx]

Supplementary materials

**Supplementary Table 1.** Odds of a household purchasing individual food groups (n 956). Eggs and other fruits were excluded because less than 4% of households reported purchasing these food groups in the last month. The models accounted for survey group, mother’s education, size of household, and clustering at the village level.

|  | **Staples** | **Pulses** | **Seeds** | **Dairy** | **Flesh foods** | **DGLV** | **Other Vit. A** | **Other Veg^1^** |
| --- | --- | --- | --- | --- | --- | --- | --- | --- |
| Within 30 minutes of a market | 1.53^**^ | 1.08 | 1.03 | 1.2 | 1.6 | 1.49^*^ | 1.75^*^ | 0.95 |
|  | [1.11,2.10] | [0.76,1.52] | [0.64,1.64] | [0.82,1.77] | [0.96,2.67] | [1.09,2.05] | [1.09,2.80] | [0.60,1.51] |
| High market food diversity | 1.56^*^ | 1.43 | 1.07 | 1.19 | 0.91 | 1.16 | 0.89 | 1.39 |
|  | [1.06,2.32] | [0.93,2.19] | [0.61,1.87] | [0.78,1.83] | [0.44,1.88] | [0.83,1.61] | [0.37,2.13] | [0.80,2.41] |
| Poorest Quintile | Ref. | Ref. | Ref. | Ref. | Ref. | Ref. | Ref. | Ref. |
| Second | 1.06 | 1.23 | 1 | 0.92 | 0.85 | 0.96 | 1.23 | 1.39 |
|  | [0.67,1.67] | [0.75,2.01] | [0.47,2.12] | [0.52,1.64] | [0.44,1.61] | [0.61,1.50] | [0.66,2.30] | [0.69,2.81] |
| Middle | 1.04 | 1.1 | 1.39 | 1.32 | 0.68 | 0.92 | 1.04 | 1.36 |
|  | [0.66,1.64] | [0.66,1.82] | [0.68,2.83] | [0.76,2.30] | [0.35,1.32] | [0.58,1.45] | [0.55,1.97] | [0.68,2.74] |
| Fourth | 1.05 | 1.29 | 1.55 | 1.05 | 1.56 | 1.18 | 1.06 | 0.89 |
|  | [0.66,1.68] | [0.78,2.14] | [0.76,3.14] | [0.59,1.86] | [0.73,3.33] | [0.75,1.85] | [0.56,2.03] | [0.42,1.90] |
| Least Poor | 0.58^*^ | 0.77 | 2.11^*^ | 1.06 | 1.73 | 1.06 | 1.03 | 1.65 |
|  | [0.35,0.95] | [0.44,1.34] | [1.01,4.42] | [0.57,1.95] | [0.76,3.97] | [0.65,1.72] | [0.51,2.10] | [0.79,3.44] |
| Missing info | 1.07 | 1.81 | 3.71 | 3 | 0.7 | 0.75 | 1.94 | 1.63 |
|  | [0.34,3.38] | [0.50,6.54] | [0.79,17.54] | [0.77,11.64] | [0.10,5.05] | [0.25,2.27] | [0.41,9.13] | [0.33,8.04] |
| Yearly expenditures (million Tanzanian shillings) | 1.14^*^ | 1.13^*^ | 1.14 | 1.11 | 0.98 | 0.99 | 1.11 | 1.15^*^ |
|  | [1.03,1.27] | [1.02,1.25] | [1.00,1.30] | [0.99,1.24] | [0.84,1.14] | [0.90,1.09] | [0.97,1.26] | [1.01,1.30] |

DGLV, dark green leafy vegetables. Vit, vitamin ^1^Denotes vegetables that are not rich in Vitamin A. *P<0.05, **P<0.01, ***P<0.001
